# Hydrothermal Modification of Wood: A Review

**DOI:** 10.3390/polym13162612

**Published:** 2021-08-06

**Authors:** Md. Rowson Ali, Ummi Hani Abdullah, Zaidon Ashaari, Norul Hisham Hamid, Lee Seng Hua

**Affiliations:** 1 Department of Natural Resources Industry, Faculty of Forestry and Environment, Universiti Putra Malaysia, Serdang 43400, Malaysia; rowson_ali@yahoo.com (M.R.A.); zaidon@upm.edu.my (Z.A.); h_noroul@upm.edu.my (N.H.H.); 2 Seasoning and Timber Physics Division, Bangladesh Forest Research Institute, Chattogram 4217, Bangladesh; 3 Institute of Tropical Forestry and Forest Products (INTROP), Universiti Putra Malaysia, Serdang 43400, Malaysia

**Keywords:** hydrothermal modification, buffered media, thermal treatment, wood modification, dimensional stability, strength properties

## Abstract

Wood is a versatile material that is used for various purposes due to its good properties, such as its aesthetic properties, acoustic properties, mechanical properties, thermal properties, etc. Its poor dimensional stability and low natural durability are the main obstacles that limit its use in mechanical applications. Therefore, modification is needed to improve these properties. The hydrothermal modification of wood exposes wood samples to elevated temperatures and pressure levels by using steam, water, or a buffer solution as the treating medium, or by using superheated steam. Abundant studies regarding hydrothermally treated wood were carried out, but the negative effect on the wood’s strength is one of the limitations. This is a method that boosts the dimensional stability and improves the decay resistance of wood with minimal decrements of the strength properties. As an ecofriendly and cost-effective method, the hydrothermal modification of wood is also a promising alternative to conventional chemical techniques for treating wood. Researchers are attracted to the hydrothermal modification process because of its unique qualities in treating wood. There are many scientific articles on the hydrothermal modification of wood, and many aspects of hydrothermal modification are summarized in review papers in this field. This paper reviews the hydrothermally modified mechanical properties of wood and their potential applications. Furthermore, this article reviews the effects of hydrothermal modification on the various properties of wood, such as the dimensional stability, chemical properties, and durability against termites and fungi. The merits and demerits of hydrothermal wood modification, the effectiveness of using different media in hydrothermal modification, and its comparison with other treating techniques are discussed.

## 1. Introduction

Wood is considered to be an important biomass that is used for multiple purposes due to its high strength value. Demand for its utilization is increasing, which is mainly due to the increase in the global population. However, forest-covered areas are simultaneously decreasing. FAO reports [1] categorized global forest products into industrial roundwood, sawnwood, wood-based panels, fiber furnish, paper and paperboard, wood fuel, charcoal, and pellets. Global industrial roundwood removals amounted to about 2028 million m^3^ in 2018. The top five countries (USA, Russia, China, Brazil, and Canada) produced 53% of the total production of roundwood removals. Industrial roundwood is roundwood that is used for any kind of purpose, and it comprises, for example, pulpwood, sawlogs, and veneer logs. Wood’s dimensional instability and nondurable nature bind it to limited uses compared to plastic or synthetic materials. Wood is also porous and hygroscopic in nature, and it contains cell walls. Wood is degradable and highly susceptible to biological degradation by termites, fungi, and insects. Some wood is less durable because of the high carbohydrate content stored in parenchymal cells and the lack of toxic extractives. According to Stirling et al. [2], wood is also susceptible to a variety of wood-deteriorating agents, especially *Basidiomycota fungi* (white rot and brown rot) and *Ascomycota fungi* (soft rot and stain). Termites, mold, bacteria, algae, and lichens also cause massive alterations to wood. The presence of these types of deteriorating agents in a wooden structure can destroy wood-made products within a short period of time, causing financial losses and negatively impacting the environment [3]. Therefore, modification helps to reduce the hygroscopic nature of wood, which is needed for the improvement of its dimensional stabilization and to prevent biological deterioration agents’ attacks on wood.

Thermal or heat treatment is an apex method used to modify wood [4]. According to Hill [5], wood modification is a process that improves some wood properties without environmental hazards. In the heat treatment process, wood is generally heated to temperatures ranging from 180 to 260 °C, where lower temperatures cause minor changes in wood constituents, while higher temperatures cause severe changes [5]. Kocaefe et al. [6] reported that the thermal treatment of wood is also conducted at higher temperatures (150–240 °C) than those of conventional drying techniques. However, some research on wood showed that a lower temperature (100 °C) can degrade wood constituents depending on the species used and the desired material properties [7,8,9]. The reduction in wood hygroscopicity was first proved by Tiemann [10], where moisture-sorption reduction occurred at 10–25% when the wood was treated with steam at 150 °C for 4 h.

In order to change the chemical structure of wood, novel methods have had renewed attention. These alteration processes aim to improve various properties of wood, such as a reduction in the emission of volatile organic compounds [11], stress reduction [12,13,14], improved dimensional stability, color uniformity, and durability [13,15]. The different chemical changes invariably lead to changes in the various physical properties of wood, but also reduce its strength properties [13]. The decrement in strength is one of the main weaknesses for the commercial utilization of thermally treated wood. In addition, the thermal treatment of wood can be performed in different treating media, such as air, water or steam, and nitrogen. Every medium can affect the wood properties differently.

Nowadays, the hydrothermal modification of wood in different media is one of the most prominent methods among the thermal treatment techniques [16,17]. The notion of hydrothermal modification can be largely defined on the basis of a variety of temperatures and pressures [18]. In addition, it can be performed in the presence of steam or liquid water under pressure or vacuum at various temperature levels, and it can affect the various characteristics of wood. Although there is a variety of hydrothermal conditions, this review focuses on the hydrothermal modification of wood in which water and other buffer solutions serve as treating media. 

Hydrothermal modification reduces the equilibrium moisture content (EMC) of treated wood samples, which subsequently leads to the enhancement of the dimensional stability. The reduction in the EMC is caused by heat-induced factors, namely, reducing hydroxyl groups [19], and it is considered to be an eco-friendly and cost-effective method because no chemicals are used [20]. In hydrothermal modification in buffered media, treated wood is more hydrophobic in nature, as the absorption of water into wood is limited and may prevent fungal growth. It also has improved decay resistance [21] and minimal decrements of strength properties [22,23]. There are many types of wood modification methods that enhance wood properties; for example, heat, oil heat, chemical treatment, and hydrothermal modification. Review works on different modification methods have already been published. A comprehensive review on wood modification by heat treatment was conducted by Esteves and Pereira [24]. Another review by Sanberg et al. [25] comprehensively discussed the development of three types of wood modification processes—namely, chemical processing, thermo-hydro processing, and thermo-hydro-mechanical processing. Their environmental impacts were assessed with a life-cycle assessment (LCA). The thermal treatment of wood by using vegetable oils was reviewed by Lee et al. [26]. In addition, the hygroscopic and dimensional behavior of thermally modified timber treated under dry and wet conditions was also critically reviewed by Hill et al. [27]. The effects of chemical modification on the strength properties of wood and the effects of heat treatment on its dimensional stability were reviewed by Xie et al. [28] and Kokaefe et al. [29]. The main objective of the present study is to review published articles about wood properties altered through hydrothermal modification in different media and to find appropriate treatment conditions for improving wood modification.

## 2. Wood Modification

**Figure 1:**
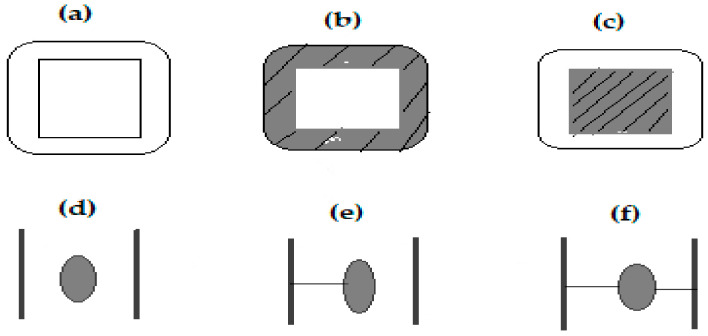
Different types wood modification at the cellular level [30].

Wood modification is an important all-around term regarding the application of different methods (chemical, physical, or biological) to alter the poor properties of wood by altering its chemical constituents to produce a new material that is environmentally friendly. According to Hill [5], wood modification encompasses the action of a chemical, biological, or physical agent upon the material that results in the augmentation of a desired property during the service life of the modified wood. Further, modified wood should be nontoxic and should not release any harmful substances during service. Furthermore, wood modification comprises two phases—active and passive. The active phase results in chemical changes that are derived from chemical, thermal, and enzymatic modifications. On the other hand, the passive phase does not produce chemical changes, but could produce an outcome for the physical properties, such as impregnation modification. The types of wood modification processes are shown in Figure 1, and the classifications of different types of wood modification are presented in Table 1.

## 3. Hydrothermal Modification of Wood

Generally, hydrothermal modification is performed in the presence of steam or liquid water under pressure at various temperature levels. The typical treatment temperature ranges from 180 to 260 °C [26]. As only water is used as the treatment medium, no hazardous chemicals are used. Therefore, among the wood modification processes, hydrothermal modification is the most commercially advanced technique for improving some of the properties of wood [5]. It is an alternative way of treating wood without using chemicals. 

Hydrothermal treatment of wood can be generally classified into three categories—namely, supercritical water treatment, subcritical water treatment, and ambient liquid-water treatment [18]. The difference between these methods is the temperature applied during the treatment. Supercritical water treatment involves a treatment temperature of more than 374 °C, while subcritical and ambient liquid-water treatments involve treatment temperatures of 100 to 374 °C and 25 to 100 °C, respectively. Most of the published literature falls within the category of subcritical water treatment, where saturated steam is commonly used to hydrothermally modify wood. However, this review emphasizes the hydrothermal modification of wood that takes place at 100 to 374 °C with water and other buffer solutions as treating media. The treatment is normally carried out in a confined high-pressure tank filled with water. The wood samples are submerged in the water or buffered solutions (acidic, neutral, or alkaline), and the solution is heated up to the desired temperature, which is maintained for a desired period of time. 

Wood properties can be considerably enhanced by converting hydrophilic OH– groups into more hydrophobic groups and by reducing the equilibrium moisture content. In addition, when hydrothermal modification is performed in buffered media, hydroxyl groups can also be removed from the cell wall because, when using water as the treatment medium, there are no more bonding sites for water molecules.

**Figure 2:**
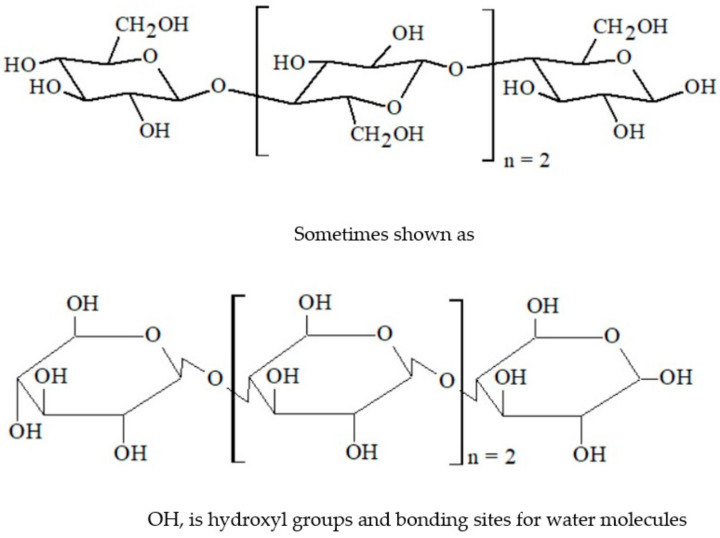
Cellulose structure in the cell walls of wood [33].

Figure 2 illustrates that, if hydroxyl groups (OH–) are removed, there are no more bonding sites for water molecules. Therefore, cell-wall swelling is reduced. Moreover, hydrothermal modification in buffered media (acidic, neutral, and alkaline) can play a vital role in enhancing some wood properties—namely, physical, strength, chemical, and biological. However, it has some drawbacks, as it decreases the pH, which leads to a mass-loss increase and subsequent decline in wood strength [21,22,31]. Another drawback is the increased water absorption (WA) with increasing treatment duration and temperature [32].

Mohebby and IIbeighi [34] reported a positive effect of thermal water treatment on the dimensional stability of *Morus alba* wood due to the decrease in the wood hygroscopicity due to chemical changes at high temperature levels; the dimensional stability of the wood under the thermal treatment mainly increased. 

Talaei [35] studied the effects of hydrothermal modification in a buffered medium on the natural durability and physicomechanical properties of beech wood (*Fagus orientalis*) in Iran. She reported that lower densities were observed in an acidic medium because of the higher degradation of hemicelluloses in the acidic medium. The mass loss in neutral and alkaline media was lower because of the loss of carbohydrates being degraded. There was a direct relation between the higher degradation and lower density of the specimens. Mass loss in wood is one of the important features of hydrothermal modification. The variations in mass loss during thermal treatment depend on the wood species, acidity (pH) of the treatment medium, temperature, and time [36].

Ebadi et al. [37] conducted a study on the physical behavior of hydrothermally treated oil palm wood (*Elaeis guineensis* Jacq.) in different buffered pH media. This study investigated seven parameters: wood density, equilibrium moisture content (EMC), mass loss (ML), water absorption (WA), volumetric swelling (S_V_), anti-swelling efficiency (ASE), and water-repellent efficiency (WRE). The results revealed that the EMC and treatment-induced mass loss of the samples were lower in the alkaline buffered solution (pH 8) compared to those in the acidic buffered solution (pH 5). It was reported that the alkaline buffered solution was able to neutralize the negative effects caused by acid hydrolysis at high temperatures. Consequently, the oil palm wood that was thermally modified in alkaline conditions possessed better ASE and WRE. 

In addition, Ebadi et al. [38] examined the effects of hydrothermal modification on oil palm wood in buffered media with different pH. They focused on evaluating the mechanical properties of the modulus of rupture (MOR), modulus of elasticity (MOE), compression parallel to grain (PC_II_), and hardness strength for treated and untreated samples. They also found that, for oil palm wood, buffering the hydrothermal modification in the weak range of alkaline media allowed a better performance in preventing a decline in mechanical strength compared to the other media used, such as acidic media and water, and they suggested this for structural applications. Saliman et al. [39] investigated the effect of hydrothermal modification on oil palm wood in terms of decay resistance against white-rot fungus and equilibrium moisture content. The evaluation of the results showed that a slightly better decay resistance was achieved when the samples were treated in neutral and alkaline media compared to acidic media. The EMC of the treated samples was reduced with increasing treatment temperatures. A lower EMC was recorded in the samples treated in the acidic medium due to the accelerated decomposition of holocellulose in the acidic conditions. In addition, an improvement in decay resistance was also recorded in line with the reduction of the EMC. No significant differences in term of decay resistance were observed between the samples treated in alkaline and acidic conditions. However, the oil palm wood treated in neutral and alkaline media did show slightly better decay resistance. The authors suggested that this may have been caused by the fact that the samples treated in acidic conditions had a higher lignin content, which is a preferred food source for white-rot fugus. Hydrothermal modification in different buffered media can therefore enhance dimensional stability and biological durability with minimal decrements in the strength properties of wood. The advantages and disadvantages of hydrothermal wood modification are shown in Table 2.

Apart from that, darkening in wood color was also a typical observation for wood that underwent hydrothermal treatment. The changes in color are possibly due to the production of chromophores as a result of hemicellulose degradation at high temperatures [26]. The extent of wood darkening is a function of the treatment temperature and time. The treated wood becomes darker with increasing treatment temperature and time [39]. 

## 4. Variables Determining Hydrothermal Modification Performance

The efficacy of hydrothermal modification mostly depends on the heating medium, duration, temperature, and wood species used. When thermal treatment is carried out in the presence of steam, water, or acidic, neutral, or alkaline media, the wood properties are different [22,37,38].

### 4.1. Medium

The effect of hydrothermal modification on wood properties mostly depends on the treatment medium. Woods treated in different media (steam, water, acidic buffer, neutral buffer, or alkaline buffer) exhibit different properties. Some of these, especially the physical properties, were found to be superior in acidic and water media, and some, especially the mechanical and biological properties, were better in neutral and alkaline media.

A study on the response surface modeling of the hydrothermal modification conditions with respect to the color changes, strength, and durability properties of rubberwood was conducted by Wongprot et al. [40]. They used water as the heating medium and investigated several parameters, such as the EMC, tensile strength, shear strength, and mass loss caused by fungal and termite attacks. The study reported an improvement in durability against both wood deterioration agents, but also showed that the tensile and shear strength were simultaneously hampered. Ebadi et al. [37] found that the dimensional stability of oil palm wood was better in acidic media than that treated in alkaline media. The mechanical properties of beech wood showed smaller decrements in neutral and alkaline media than those in water and acidic media, and this varied with temperature [22]. Better decay resistance was also recorded in oil palm wood that was modified in neutral and alkaline media compared to the samples modified in an acidic medium [39]. So, the effectiveness of hydrothermal modification mostly depends on its treatment medium.

### 4.2. Wood Species

The physical, mechanical, chemical, and biological properties of wood vary with age, anatomical features, and location. Different wood species exhibit different properties. Some wood species are unstable, light, nondurable, and weak, while others have medium or high strength. On the basis of these properties, wood can be used for various purposes. Several treatment methods, such as heat, chemical, or oil-heat treatment and hydrothermal modification, are applied to enhance the properties of wood. Hydrothermal modification can differently affect temperate and tropical hardwoods. It also affects the physical, mechanical, chemical, and structural properties of wood while influencing the moisture diffusion coefficient during drying [41]. Studies on the hydrothermal modification of tropical hardwoods are relatively minimal in comparison with those on temperate hardwoods. Generally, tropical hardwoods differ from temperate hardwoods in terms of chemical composition and anatomical structure.

Varga et al. [42] examined the hydrothermal modification of two European hardwood species—black locust (*Robinia pseudoacacia*) and oak (*Quercus robus*)—and two tropical hardwood species—mearbau (*Intsia bijuga*) and sapupira (*Hymenolobium petracum*). This research investigated the effects of hydrothermal modification with different temperature levels and treatment times on the physical and mechanical properties of these species. The study found that black locust and oak were much more sensitive to hydrothermal modification than the tropical species were. The results also showed that the bending strength of treated samples, particularly the black locust and oak species, was highly influenced by the treatment time and temperature. The reduction in the bending strength of oak was remarkable even at low temperatures. De Freitas et al. [43] investigated the effects of hydrothermal modification on *Eucalyptus grandis* wood and revealed that hydrothermal modification at 140 °C for 25 min promoted changes in some properties, such as a reduction in hygroscopicity and the homogenization of superficial color, without the loss of mechanical resistance. In addition, Rasdianah et al. [44] reported that the superheated-steam treatment of two tropical hardwood types—namely, light red meranti (*Shorea* spp.) and kedondong (*Canarium* spp.) wood—caused them to exhibit lower equilibrium moisture content and decreased strength properties. On the basis of the above discussion, hydrothermal modification can affect temperate and tropical hardwoods differently because of their different chemical compositions and anatomical features.

## 5. Effects of Hydrothermal Modification on Wood Properties

Hydrothermal modification affects the properties of treated wood. Table 3 lists some studies that reported the effects of hydrothermal modification on wood properties.

### 5.1. Morphology

Wood is a heterogeneous biological material, and its cell walls’ chemical composition primarily comprises cellulose, hemicellulose, lignin, and extractives. Moreover, the wood microstructure is characterized by the multiformity of its cell elements. Changes in the wood microstructure occur during hydrothermal modification [47,48,49,50]. Hydrothermal modification alters the ultrastructure and pore characteristics of wooden materials. Wood is porous, with micropores, mesopores, and macropores in various proportions. Wood porosity depends on the density and anatomical features of the wood species. 

Biziks et al. [51] conducted a study on birch wood (*Betula pendula*), and samples were treated in a thermal regime (140, 160, and 180 °C) for 1 h. The results revealed microstructural changes by means of scanning electron microscopy (SEM), and significant changes in the linear size of birch wood were also observed. The analysis also showed that fibers formed a major part of the wood and acted as the principal effect on its total structural changes following modification. Treated wood (180 °C) showed that the cross-sectional and wall areas and the wall thickness of the fibers were reduced by 21%, 37%, and 32% on average, respectively; voids and cracks were also formed between the fibers. 

Zhang and Cai [52] revealed that 6% to 13% of steam-treated (160 °C) sub-alpine fir (*Abies lasiocarpa* (Hook.) Nutt.) collapsed during post-treatment kiln drying, which indicated that the treatment significantly altered the cell-wall structure. Further, de Freitas et al. [43] reported that hydrothermal modification promoted the partial cleaning of the elements (vessels). Shi et al. [53] hydrothermally treated poplar wood (*Populus* sp.) in water, diluted acid (H_2_SO_4_), and diluted alkali (NaOH) aqueous solution and reported that the compound middle lamellas of wood cells were damaged after treatment. Poplar wood treated in diluted acid aqueous solution exhibited the greatest extent of damage, where both the middle lamella layers and the secondary cell wall were severely destroyed. Meanwhile, the poplar wood that was treated in water and diluted alkali aqueous solution only showed fractures in the middle lamella layers. The crushing of the cellular lumen of radiata pine (*Pinus radiata* D. Don) wood was observed after thermal treatment, and the extent of crushing increased with increasing treatment temperature [54]. Furthermore, Wang et al. [55] reported that the diameters of fibers of Chinese sweet gum (*Liquidambar formosana* Hance) did not have significant changes, and the wall–cavity ratio only changed from 0.38 to 0.41 due to the medium–low-temperature hydrothermal treatment. 

### 5.2. Physical Properties

Hydrothermal modification can affect the physical properties of wood—namely, the equilibrium moisture content (EMC), density, mass loss (ML), volumetric swelling coefficient (VSC), and water absorption (WA). When wood is subjected to thermal treatment, formic and acetic acids are formed, and they cause the degradation of hemicellulose [56]. Hydrothermal modification can improve the dimensional stability by substantially altering the chemical composition of wood. On the other hand, density is a very important factor of wood properties, and it is affected by mass loss during hydrothermal modification. During hydrothermal modification, mass loss depends on the wood species, heating medium, temperature, and duration [24]. 

Scheiding et al. [57] carried out a study on hydrothermal modification at 190 and 210 °C for 3 h on Scots pine (*Pinus sylvestris*). This study exhibited significantly different water-absorption characteristics in the treated pine wood. 

Hydrothermal modification reduces the equilibrium moisture content (EMC) of treated wood samples, which consequently leads to enhanced dimensional stability. The EMC can be reduced by various heat-induced factors, such as a diminishing amount of water-affinity hydroxyl groups [19]; the unavailability of hydroxyl groups to water molecules is due to the increase in the crystallinity of cellulose [36,58]. Charani et al. [33] carried out a study on the influence of hydrothermal modification on the dimensional stability of beech wood. This study investigated the effects of hydrothermal treatment on dimensional stability, density, and water absorption. They found that the best anti-swelling efficiency was observed at 170 °C for 1 h among the different treatment temperatures and durations. This modification process also exhibited a slight decrease in specific gravity at 150 to 170 °C.

Kartal et al. [59] stated that the water absorption of specimens treated at high temperature levels with boron compounds increased with treatment temperature and time. Salim et al. [60] reported that steaming and compression at a high temperature enhanced the physical properties and dimensional stability of intensive OPT. Rahimi et al. [45] reported that hydrothermal modification (140 °C) significantly changed the total porosity of yellow poplar (*Liriodendron tulipifera* Linnaeus) samples, and that hydrothermal modification significantly increased water absorption for all treatments except hot compressed water at 100 °C.

### 5.3. Mechanical Properties

The mechanical or strength properties of wood are affected by hydrothermal modification due to the heat-induced alteration of the chemical composition of cell-wall components (cellulose, hemicellulose, lignin, and extractives). During hydrothermal processes, the released acids cause a decrease in pH, the deacetylation of hemicellulose, mass loss, and, consequently, the reduction of mechanical strength [32,61]. Theander and Nelson [62] stated that the degradation rate of carbohydrates is high in acidic situations and is promoted by the high availability and low crystallinity of hemicelluloses. Further, variations in the acidity of the treatment media increase due to thermal treatment in wet environments because of the formation of acetic and formic acids on the basis of the decomposition of hemicellulose during hydrothermal modification in acidic media [63,64]. In addition, Kim et al. [65] stated that starch, hemicellulose, and extractives are degraded due to thermal treatment in acidic hydrolysis. Poncsak et al. [66] investigated the effects of high treatment temperature on the strength properties of birch wood. They reported that the modulus of elasticity (MOE) and modulus of rupture (MOR) decreased with increased treatment temperature. Dundar et al. [46] reported that the MOE and MOR of hydrothermally treated (180 °C) black pine wood showed a significant reduction. 

Mirzaei et al. [67] conducted a study on the effects of hydrothermal treatment on the bond shear strength of beech wood. The results revealed that both the bond shear strength and shear strength parallel to the grain decreased with increasing hydrothermal treatment temperature due to the hydrophobic behavior of the wood surface as a result of the treatment. The decreased wettability of the wood surface negatively affected the penetration of adhesive and, therefore, weakened the bondline. 

Talaei et al. [22] carried out a study on the hydrothermal modification of beech wood in buffered media. They explored the parameters of compression strength parallel to the grain, shear strength, and hardness. The analysis revealed that the pH (7 and 8) neutralized the released acids, and the pH was a fixed at a neutral level. Buffering the medium of hydrothermal modification at a neutral level prevented strength loss and extended the usage of heat-treated beech (*Fagus orientalis*) wood in load-bearing applications.

Rasdianah et al. [44] reported that the MOR of both thermally modified light-red meranti (*Shorea* spp.) and kedondong (*Canarium* spp.) wood was reduced compared with that of untreated samples. The authors also mentioned that the MOE of the thermally modified samples was reduced with increased treatment temperature and time. Rahimi et al. [45] investigated the effects of different hydrothermal modifications (steam and hot compressed water) on the physical properties and drying behavior of yellow poplar (*Liriodendron tulipifera*). They found that hydrothermally treated samples (100 and 140 °C) exhibited a greater modulus of elasticity than that of the control samples.

### 5.4. Chemical Properties

Wood is a nonuniform biological material that contains a variety of cells. Its cell walls are composed of cellulose, hemicellulose, lignin, and small amounts of extractives, proteins, and inorganic components depending on the height and species [68]. These main wood components experience different extents of changes due to heat treatment. For instance, hemicellulose degrades through deacetylation, depolymerization, and dehydration, while cellulose exhibits increased crystallinity. Meanwhile, some structural changes occur in lignin due to polycondensation reactions and crosslinking with cell walls. Most of the extractives disappear or degrade, particularly those of volatile ones, but new compounds can also be formed. Different ratios of the chemical structure influence wood characteristics differently, even within the same species. This is due to the different functions of chemical compounds in wood that affect its properties [69]. During hydrothermal modification, the acetic acid formed by the acetyl groups of hemicellulose increased the pH of the treatment medium [70]. Sik et al. [71] reported that the hygroscopicity of rubberwood treated in the temperature range of 100 to 150 °C was reduced by the increased wood crystallinity and the reduction in hydroxyl groups within the cell walls. 

Hydrothermally treated wood in an acidic buffer medium leads to an increase in the crystalline index and a higher rate of lignin. Neutral and alkaline buffers, on the other hand, considerably control carbohydrate destruction [72]. Sundqvist [32] reported that the original sources of the weak acid (formic acid) released during hydrothermal modification of birch wood are formate esters, and the sources of acetic acid are acetate esters from methyl-glucuronoxylan. Yildiz and Gümüskaya [73] reported an increase in cellulose crystallinity through heat treatment. Mitsui et al. [74] reported that the degradation of hydroxyl groups in cellulose is amorphous, semicrystalline, or crystalline. Inagaki et al. [75] reported an increase in cellulose crystallinity and crystallite thickness due to hydrothermal modification because of the destruction of the amorphous part of cellulose and hemicellulose; they also showed a good correlation between average crystallite size and the level of reduced availability of the crystalline regions. 

Furthermore, Cai et al. [76] conducted a study on the effect of pressurized hot water treatment on the mechanical properties, surface color, chemical composition, and crystallinity of pine wood. They revealed that the lignin percentage ratio was increased and bending strength was decreased after hydrothermal modification. This mainly happened because of the degradation of hemicelluloses and other products. Lignin degradation occurred only when the treatment temperature was above 200 °C.

### 5.5. Biological Properties

The biological properties of wood are prominent and can be modified through hydrothermal modification. There are many reasons for the improvement of the biological durability of thermally treated wood. The wood becomes more hydrophobic in nature due to the thermal modification, thus limiting the absorption of water into the wood and potentially preventing fungal growth [13]. Further, the improved decay resistance could be attributed to the reduction in the EMC in treated wood samples, which is directly proportional to the increase in temperature [77]. The hygroscopicity-reduction behavior reduces moisture retention in the treated wood and leads to improvements in fungal resistance compared to that of untreated wood samples [78]. 

Nabil et al. [79] reported that, when oil palm wood was subjected to a higher temperature, the treated wood had lower moisture absorption, which was insufficient for fungal survival and for preventing the attack of white-rot fungus. Doi et al. [80] conducted a study on heat-treated Japanese larch (*Larix leptolepis* (Sieb. et Zucc.) Gord.) and reported the improved resistance of the treated wood against *Copotermes formosanus,* but the resistance against *Reticulitermes speratus* was reduced. Hakkou et al. [81] reported a correlation between the biological durability of wood and the temperature of thermal modification. Several researchers reported that the biological properties of wood were improved due to different thermal modification methods [13,82]. The hydrothermal modification of wood can play an important role in enhancing resistance against fungal attacks. Cheng et al. [83] reported that hydrothermally treated *Moso* bamboo in different aqueous media showed better resistance against mold. Further, Endo et al. [17] revealed that hydrothermally treated Sitka spruce (*Picea sitchensis* (Bong.) Carr.) wood exhibited a trend of reductions in hygroscopicity and equilibrium moisture content (EMC). 

Saliman et al. [39] used the response surface methodology to investigate the effects of hydrothermal modification on the decay resistance of oil palm wood and found that the weight loss of the treated oil palm wood was reduced as treatment temperature and time increased. They also found that samples treated in neutral and alkaline media showed slightly better decay resistance. Militz [84] reported an increase in resistance against *Hylotrupes bajulus*, *Lyctus brunneus*, and *Anobium punctatum* for heat-treated wood. Hadi et al. [85] conducted a study on the effects of hydrothermal modification of modified poplar wood treated with acid–copper–chromate at various concentrations. They found that the improved decay-resistance properties of wood depended on the treatment temperature and time. 

## 6. Comparison of Hydrothermal Modification and Other Thermal Treatment Methods

Hydrothermal modification is an effective technique for controlling and neutralizing the destructive effects of acids formed through the destruction of carbohydrates during the treatment. The main advantage of a buffered solution is its nonchemical modification that does not pollute the environment [86]. The use of buffered media in hydrothermal modification is an eco-friendly and nonchemically modified method [14]. Hydrothermal modification makes some changes in wood through the removal of extractives, hemicellulose hydrolysis, and the transformation of the properties of lignin and cellulose [87]. 

Many authors compared the efficiencies of thermal treatment methods in terms of wood properties. In general, hydrothermal modification can be less aggressive compared to other thermal treatment methods. Treatment carried out with waterborne solutions reduces the strength properties of wood compared to treatment performed with oil-type solutions because treatment with aqueous solutions increases the rate of hydrolysis in wood. Zaidon et al. [88] carried out a study on the dimensional stability of bamboo by using hydrothermal modification with different buffered solutions (pH 5, 7, and 8) as the heating media; the results revealed that a more dimensionally stable sample was obtained with the neutral and alkaline media. Vivian et al. [89] reported that hydrothermal modification improved the permeability of wood through the clearing of scores and pores, the optimization of the drying process, and the attainment of products with lower percentages of defects. Saari et al. [90] reported that the steaming process resulted in a positive effect on the mechanical properties of oil palm trunk lumber. Further, Ebadi et al. [37] observed a greater mass loss in hydrothermally treated oil palm wood than in heat-treated black pine wood [91]. 

Numerous studies have been conducted around the world, and some of their research findings related to the properties of wood that in subjected to different modification pathways are shown in Table 4. 

## 7. Potential Applications of Hydrothermally Treated Wood 

The improvements in some properties after thermal treatment, such as resistance against decay, mold, or insects and the higher durability against moisture and ultraviolet (UV) radiation, have opened up various potential applications for thermally treated wood. However, due to its reduced strength, thermally treated wood is commonly recommended for use in nonstructural applications. One of the prominent changes after thermal treatment is the darkening of the wood color. The darkened color is favorable for indoor applications, such as flooring or furniture fronts [101]. A great majority of thermally modified wood is used in cladding, decking, window frames, garden furniture, interior furniture, cabinet, utility poles, fences, poles, posts, and packaging [102]. Each application has specific property requirements. For example, cladding, decking, and utility poles for outdoor exposure require very high resistance against weather, UV light, decay, mold, and insects. Interior furniture, on the other hand, requires a very good appearance and strength. More than 3000 tons of residues are generated during the thermal modification process of Thermowood^®^ products. The residues are generated in the forms of sawdust, shavings, and cut-offs. A small portion of this residue is used in the manufacturing of composites, such as Thermowood plastic composites (TWPCs), where thermally modified residue bestows better dimensional stability to TWPCs. Despite that, a large portion of the generated residue is incinerated for energy production [103]. Another study by Tavassoli et al. [104] demonstrated the use of hydrothermally treated wood fibers as reinforcing fillers for natural rubber biocomposites. A better dispersion of fibers in the rubber matrix was observed, as the hydrothermal treatment improved the interaction between the fibers and rubber. As a result, better tensile strength and elongation at break were recorded in the rubber biocomposites that were reinforced with hydrothermally treated wood fibers. 

## 8. Technoeconomic Challenges 

**Figure 3:**
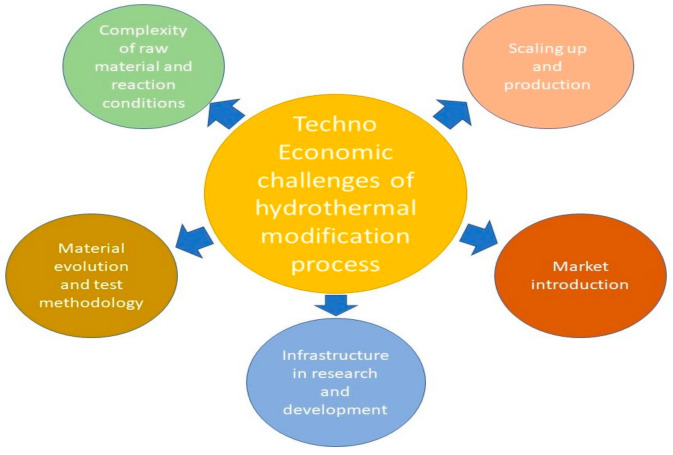
Challenges in technologies for the hydrothermal modification of wood for practical applications (summarized from Militz and Lande [105]).

Figure 3 shows the challenges in hydrothermal wood modification technologies for practical applications according to Militz and Lande [105]. The first challenge is the complexity of the raw material and reaction conditions, as wood is a very complex material. There are no two pieces of wood that are identical. Therefore, this calls for more stringent selection of materials in order to achieve the desired property changes. Promising results obtained on the laboratory scale are often not replicated when scaled up. Second, the testing methodology is another challenge. Such tests are vital for quality control and assurance. However, they can be very time-consuming and expensive. 

Provided that encouraging results are obtained on the laboratory scale, the modification method needs to be scaled up for full-sized timber and industrial equipment. This is the third challenge, where the gap is still very wide. Taking wood drying as an example, the perfect requirements and properties for drying wood were already acquired in the laboratory. However, when it came to scaling up, it still took decades before well-functioning, high-capacity wood kilns were ready for the industry [105]. Next, confidence in the market players for thermally modified wood is very crucial. Price is the main decision criterion for consumers. Thermally modified wood has increased production costs; therefore, it is of utmost importance for products to exhibit substantially increased benefits after modification. Lastly, there is a lack of existing industrial structures, particularly in some developing countries, such as Malaysia. Sadly, most studies stop at the laboratory level. In order to develop an effective modification method, Sanberg et al. [106] recommended further exploration of the chemical changes in wood for the optimization of the processing conditions. In addition, they recommended for a life-cycle and system perspective study to be conducted in order to better understand the environmental impact of hydrothermal treatment processes. Sanberg et al. [106] also suggested that the challenges being faced in scaling-up processes could be resolved by involving studies from interdisciplinary scientific domains, such as wood mechanics, wood chemistry, physics, material sciences, and numerical analysis. 

There are several globally used heat treatment processes for wood, and every process incurs different production costs. The Plato process, developed in the Netherlands, consists of a hydrothermolysis step followed by a dry-curing step. The production cost for every cubic meter of Plato wood is estimated to be at around EUR 120 (excluding the cost of timber). In France, a process called Le Bois Perdure involves artificial drying in an oven followed by heating at a high temperature in a steam atmosphere. The cost of producing wood using this process is around EUR 100 per treated cubic meter. In comparison, wood treated using other treatment processes incurs production costs that are comparable to those of hydrothermally treated wood. For example, the rectification process costs around EUR 150–160 per treated cubic meter. On the other hand, every cubic meter of oil-heat-treated wood costs around EUR 60–90 [107]. Overall, every process is unique, and the selection of the ideal treatment process is dependent on the readiness of existing infrastructures. 

## 9. Environmental Impact

According to Retfalvi et al. [108], a strong, aggressive, and acidic fluid is often left over after the modification process. This acidic fluid is a result of the condensation of moisture content from wood, which turns into steam during the heating process. This wastewater is strongly acidic, possesses high organic content, and could pose serious threats to the environment if not properly treated. The authors suggested that the wastewater could be used as a wood preservative, but its feasibility has not been tested. The thermal modification of wood is commonly recognized as an eco-friendly process, as it does not involve any chemicals. Nevertheless, proper scientific and industrial data are very hard to find. In addition, studies on the environmental impact of thermal modification processes are very scarce. Candelier and Dibdiakova [109] conducted a review of the assessments of the life-cycle impacts of the thermal-wood-treatment industry. The main environmental categories assessed were (i) resource depletion, (ii) cumulative energy intake and output, and (iii) gaseous, solid, and liquid emissions. Regarding resources, thermally treated wood offers advantages, as it encourages the usage of underutilized and low-valued wood [110]. In general, thermally modified wood could significantly contribute to environmental performance. In fact, most energy consumption and carbon dioxide emissions are during the drying process, while they are much lower during the modification process. Therefore, a better understanding of the process would reduce its ecological footprint. 

## 10. Conclusions

The hydrothermal modification of wood properties has been broadly studied. On the basis of the research findings, it is a cost-effective and environmentally friendly technique that can be used to improve wood properties. Different treatment media, temperature levels, and durations impact the wood properties differently. It also affects temperate and tropical wood species differently. The effects of hydrothermal modification on wood’s morphological, physical, mechanical, chemical, and biological properties were reported. Hydrothermal modification leads to a reduction in hydroxyl (OH–) and carbonyl groups in the cell walls of treated wood. In addition, the process can enhance the dimensional stability and decay resistance of wood without threatening the environment. Furthermore, at high temperatures (above 200 °C), the bending strength rapidly decreases; treated wood shows darker colors and becomes more brittle, and surface cracks are produced due to the further degradation of hemicellulose and the removal of OH– groups. Moreover, the minimal decrements of the strength properties of wood are apparent in hydrothermal modification with alkaline buffered media (pH 8) at lower temperature ranges (160 to 180 °C). Alkaline buffered media are a better option compared with other media (steam, water, and acidic) in thermal modification methods, and the treated wood may be used for the production of flooring and furniture, other construction purposes, and extended usage in load-bearing applications. Overall, hydrothermally treated wood offers several advantages in terms of technological performance, and it improves the properties of end products. However, these findings require a better and deeper understanding, as they involve a huge number of processing conditions. Small errors occurring in any one of these processing conditions could lead to undesired properties in the end products. In addition, studies on the environmental performance of hydrothermally modified wood are still scarce. Future studies could fill in this research gap.

## Figures and Tables

**Table 1:** Different types of wood modification [5].

Division	Types of Modification	Class	Illustration
Active	Chemical	Cell wall	(e) and (f)
		Surface	-
	Thermal	Cell wall	-
	Enzymatic	Surface	-
Passive	Impregnation	Cell-wall fill	(b) and (d)
		Lumen fill	(c)
	Untreated		(a)

**Table 2:** Advantages and disadvantages of hydrothermal wood modification.

Advantages	Disadvantages
Uses water, steam, or buffer solution as treating medium Ecofriendly and cost-effective method Minimal decrements in strength properties At lower temperature ranges, modified wood can be used for load-bearing applications	It greatly causes mass loss compared to other modification methods. Susceptible to higher temperatures Increases water absorption The smell of treated wood lasts a long time.

**Table 3:** Changes in wood properties after hydrothermal modification.

Wood Species	Treatment Condition	Modulus of Rupture (MOR; MPa)	Modulus of Elasticity (MOE; MPa)	Compression Parallel to Grain PC_II_ (MPa)	Specific Gravity	References
Yellow poplar	Hydrothermal modification at 100 and 40 °C	-	Untreated: 1290.0 Treated at 100 °C: 1170.0 140 °C: 1570.0	Untreated: 42.95 Treated at 100 °C: 38.50 140 °C: 42.27	Untreated: 0.50 Treated at 100 °C: 0.50 140 °C: 0.49	[45]
Light red meranti	Superheated steam at several temperature levels and du- rations	Untreated: 57.0 Treated at 172 °C for 180 min: 54.47	Untreated: 7482.11 Treated at 172 °C for 180 min: 6699.6	-	Untreated: 0.39 After treatment: Loss of 4.41% to 10.37%	[44]
Kedondong		Untreated: 105.88 Treated at 172 °C for 180 min: 101.58	Untreated: 11690.4 Treated at 172 °C for 180 min: 9874.8	-	Untreated: 0.62 After treatment: Loss of 4.01% to 16.23%	
Eucalyptus grandis	Hydrothermal process at 140 °C for 5 to 25 min	Untreated: 89.0 After treatment: 76.0	Untreated: 6988.0 After treatment: 6830.0	Untreated: 53.0 After treatment: 54.0	Untreated: 0.52 After treatment: 0.54	[43]
Oil palm wood	Hydrothermal modification at 140 °C for 120 min	Untreated: 39.84 After treatment: 25.15	Untreated: 5907.0 After treatment: 4866.0	Untreated: 31.87 After treatment: 8.75	Untreated: 0.57 After treatment: 0.56	[38]
Black pine	Hydrothermal modification at 180 and 210 °C	Untreated: 61.4 Treated at 180 °C: 43.0	Untreated: 5606.8 Treated at 180 °C: 4783.5	-	-	[46]

**Table 4:** Findings regarding the properties of wood subjected to different modification pathways.

Wood Species	Treatment Type	Treatment Condition	Findings	References
Beech	Hydrothermal treatment	pH 5 to 8 and temperature 160 and 180 °C for 45 min	Hydrothermal modification with pH 7 or 8 prevents the strength loss	[22]
Black locust	Heat treatment	120 °C for 24 h	Discoloration of wood due to heat treatment	[92]
Oil palm wood	Hydrothermal treatment	pH (5 and 8) and temp. 140 °C for 120 min	Enhanced physical properties	[37]
Beech wood	Heat treatment	20 to 280 °C	Improved durability of wood	[81]
Malapapaya Wood	Heat treatment	160 to 220 °C for 30 to 120 min	Decreased strength properties and increased decay resistance	[15]
Birch and aspen	Heat treatment	120 °C for 30 min	Decreased strength properties	[6]
Japanese cedar wood	Heat treatment	Temperature 170, 190, and 210 °C for 60, 120, and 240 min	Untreated MOR and MOE, 70.7 and 8100 MPa, and treated at 210 °C for 4 h, 36.0 and 6200 MPa	[93]
Rubberwood	Oil-heat treat- ment	Temp. 172 to 228 °C for 95 to 265 min	Enhanced decay resistance against *P. sanguineus*	[94]
Beech and Scots pine	Hydrothermal treatment	Temperature 165 and 185 °C	Accessible acetyl groups are cleaved	[70]
Rubber wood	Heat treatment	Temperature 100 to 150 °C	MOR and MOE values treated at 60 °C, 117.01 and 12,187 MPa, and 150 °C, 107.05 and 11,909 MPa	[95]
Norway spruce wood	Heat treatment	Temperature 113 to 271 °C	Increased cellulose crystallinity	[96]
Pine and *Eucalyptus globulus*	Heat treatment	hot air in an oven for 2–24 h at 170–200 °C and by steam in an autoclave for 2–12 h at 190–210 °C.	Both woods became darker in color	[97]
*Eucalyptus globulus*	Heat treatment	2–24 h and temperatures of 170–200 °C	Attained better dimensional sta- bility	[98]
Spruce and Beech	Heat treatment	150, 180, and 200 °C for 6 and 10 h	Changed cellulose crystallinity	[73]
Scots pine Populus nigra Birch Black pine wood	Hydrothermal treatment Heat treatment Heat treatment Heat treatment	100 to 240 °C 24 h at 45 °C, 24 h at 145 °C, and 4 h at 185 °C 120 to 230 °C 160 to 200 °C for 2 and 6 h	Lignin content increased MOR decreased from 529 to 461 kg/cm^2^ MOR decreased increased tem- perature Bending-strength values de- creased 3.2% when treated at 160 ° C for 2 h and 47.2% at 200 °C for 6 h	[99] [100] [66] [91]
Pine wood	Hydrothermal treatment	140 to 200 °C for 1 to 5 h	Relative content of lignin (%): Untreated: 28.401 Treated at 160 °C for 5 h, 31.795 Treated at 180 °C for 5 h, 33.916 Treated at 200 °C for 5 h, 40.473	[76]

## Data Availability

Not applicable.
